# Evaluation of NAFLD and fibrosis in obese patients – a comparison of histological and clinical scoring systems

**DOI:** 10.1186/s12876-020-01400-1

**Published:** 2020-08-05

**Authors:** Sophia Marie-Therese Schmitz, Andreas Kroh, Tom Florian Ulmer, Julia Andruszkow, Tom Luedde, Jonathan Frederik Brozat, Ulf Peter Neumann, Patrick Hamid Alizai

**Affiliations:** 1 Department of General, Visceral and Transplantation Surgery, RWTH Aachen University Hospital, Pauwelsstr.30, 52074 Aachen, Germany; 2 Department of Surgery, Maastricht University Medical Center, P. Debyelaan 25, 6229 Maastricht, HX Netherlands; 3 Institute of Pathology, RWTH Aachen University Hospital, Pauwelsstr, 30, 52074 Aachen, Germany; 4 Department of Gastroenterology, Digestive Diseases and Intensive Care Medicine, RWTH Aachen University Hospital, Pauwelsstr. 30, 52074 Aachen, Germany; 5 Department of Gastroenterology, Hepatology and Infectious Diseases, University Hospital Duesseldorf, Medical Faculty of the Heinrich-Heine-University, Moorenstr. 5, 40225 Duesseldorf, Germany

**Keywords:** NAFLD, Non-invasive tests, Scoring system, Liver biopsy, Steatosis, Fibrosis, APRI

## Abstract

**Background:**

Non-alcoholic fatty liver disease (NAFLD) is a frequent condition in obese patients and regularly progresses to non-alcoholic steatohepatitis (NASH) and subsequent cirrhosis. Histologic evaluation is the gold standard for grading and staging, but invasive biopsies are associated with obvious risks. The aim of this study was to evaluate different non-invasive tools for screening of NAFLD and fibrosis in obese patients.

**Methods:**

In a prospective cohort study liver specimens of 141 patients were taken during bariatric surgery. Serological parameters and clinical data were collected and the following scores calculated: NASH clinical scoring system (NCS), aspartate aminotransferase to platelet ratio index (APRI), FIB-4 as well as NAFLD fibrosis score (NFS). Liver function capacity was measured preoperatively by LiMAx test (enzymatic capacity of cytochrome P450 1A2). Intraoperative liver biopsies were classified using NAFLD activity score (NAS) and steatosis, activity and fibrosis (SAF) score.

**Results:**

APRI was able to differentiate between not NASH and definite NASH with a sensitivity of 74% and specificity of 67% (AUROC 0.76). LiMAx and NCS also showed significant differences between not NASH and definite NASH. No significant differences were found for NFS and Fib-4. APRI had a high sensitivity (83%) and specificity (76%) in distinguishing fibrosis from no fibrosis (AUROC = 0.81). NCS and Fib-4 also revealed high AUROCs (0.85 and 0.67), whereas LiMAx and NFS did not show statistically significant differences between fibrosis stages. Out of the patients with borderline NASH in the histologic NAS score, 48% were classified as NASH by SAF score.

**Conclusions:**

APRI allows screening of NAFLD as well as fibrosis in obese patients. This score is easy to calculate and affordable, while conveniently only using routine clinical parameters. Using the NAS histologic scoring system bears the risk of underdiagnosing NASH in comparison to SAF score.

## Background

Non-alcoholic fatty liver disease (NAFLD) is a common disease with a steadily rising prevalence [1]. In the Western world, studies report a prevalence of 10–30%, depending on the modality of diagnosis. NASH has advanced to be the leading cause for elevated liver enzymes in routine measurements [2, 3]. The progressive form of NAFLD is called non-alcoholic steatohepatitis (NASH), which potentially leads to fatal conditions such as cirrhosis and hepatocellular carcinoma [3–5]. Risk factors for developing NAFLD include age, ethnicity and metabolic conditions such as diabetes and obesity [2]. Prevalence is therefore particularly high in obese people undergoing bariatric surgery (50–90% NAFLD and 10–50% NASH) [6–8]. The worldwide epidemic of obesity and the metabolic syndrome make NAFLD the most common cause of chronic liver disease and the second leading etiology among adults awaiting liver transplantation in the United States [2, 9].

Liver biopsy has remained to be the gold standard for diagnosing NAFLD and NASH, but such invasive procedures are neither affordable nor indicated in the majority of patients at risk for developing NAFLD [10]. Non-invasive scoring systems have thus been established to diagnose NAFLD [11–16]. The AASLD practice guidelines suggest the use of non-invasive tools to aid clinical decision, e.g. NAFLD fibrosis score (NFS), FIB-4, aspartate aminotransferase to platelet ratio index (APRI) [17]. Out of these scores, only the NFS has been developed for usage in patients with NAFLD [14].Fib-4 was originally designed for prediction of fibrosis in patients with HIV/HCV coinfection, while APRI was introduced for patients with chronic hepatitis C [11, 16, 18]. NAFLD can also be detected by a liver function capacity test (LiMAx) [19], which was initially used for predicting postoperative outcome after liver resections [20]. The aim of this study was to compare the accuracy of various non-invasive tests to predict NAFLD and fibrosis in obese patients undergoing bariatric surgery.

## Methods

### Study design

This study was designed as a prospective cohort study. All participants were candidates for bariatric surgery between 2013 and 2018 and had either body mass indices of > 40 kg/m^2^ or > 35 kg/m^2^ with weight-related co-morbidities. Patients with a history of heavy smoking (> 15 cigarettes per day), alcohol consumption (> 20 g/day), age < 18 years or causes of liver disease other than NAFLD (e.g. viral hepatitis, autoimmune hepatitis) were excluded. In a secured database, clinical data (age, body weight, body height and comorbidities), liver function and biochemical parameters were saved. For diagnosis of diabetes either fasting glucose > 126 mg/dl or 2-h-plasma glucose > 200 mg/dl were used in accordance to WHO guidelines. Liver biopsies were taken from the left liver lobe during bariatric surgery as described below. Each participant provided informed, written consent prior to enrolment. Some data from this study cohort has previously been reported in another study [19]. This study was conducted in accordance with the 1964 Declaration of Helsinki and its later amendments. Ethical approval was obtained from the Ethics Committee of the Medical Faculty of RWTH Aachen University (EK 312/11).

### Laboratory tests

Blood samples were collected maximum 2 weeks prior to surgery following overnight fasting. Biochemical parameters were determined at the Institute of Clinical Chemistry. Normal range of alanine aminotransferase (ALT) and aspartate aminotransferase (AST) is < 50 U/L. Normal range of platelet is 150–400/nL and normal range for albumin is 3.5–5.2 g/L.

### Liver histology

All liver specimens were performed as wedge resections of the left lobe. Histologic specimens were reviewed by a single pathologist experienced in evaluation of liver specimens. Specimens were only evaluated after anonymization. Histologic steatosis, lobular inflammation, hepatocellular ballooning and fibrosis were evaluated according to NAFLD activity score (NAS) and steatosis-activity-fibrosis (SAF) score [21, 22]. For NAFLD activity score, specimens were classified as ‘not NASH’, ‘borderline’ and ‘definite NASH’. All specimen were additionally classified according to the SAF score as ‘no NAFLD’, ‘NAFLD’ and ‘NASH’. Fibrosis was subdivided into four stages (F1-F4) [21].

### NASH clinical scoring system

The NASH clinical scoring system was developed to predict the risk of NASH in morbidly obese people. It consists of arterial hypertension, type 2 diabetes mellitus, AST-elevation > 27 U/L, ALT-elevation > 27 U/L, sleep apnea (each awarded one point) and ethnicity (non-black: 2 points) [13].

### Liver function capacity

Capacity of liver function was measured via LiMAx test. This test is based on ^13^C-methacetin (Euriso-top, Saint-Aubin Cedex, France) metabolism by the cytochrome P450 1A2 system (CYP1A2). Two mg/kg body weight ^13^C-methacetin is injected intravenously and metabolization into acetaminophen and turnover of ^13^CO_2_ then measured as a quotient of exhaled ^13^CO_2_ to ^12^CO_2_. Analysis is performed by online breath sampling with real-time point-of-care analysis by a laser-based nondispersive isotope-selective infrared spectroscope (FLIP2, Humedics, Berlin, Germany). The test was taken during the 2 weeks prior to surgery and after an overnight fast. To ensure comparability, LiMAx test was performed on the same day as laboratory values were taken. A measured liver function capacity of > 315 μg/kg/h is considered physiological [20, 23].

### APRI

APRI score is calculated by dividing aspartate aminotransferase (AST) serum with platelet levels. A value of > 1.5 is able to predict liver fibrosis with a positive predictive value of 89%, while values of < 0.5 exclude liver fibrosis in 80% [12, 16].

### Fib-4

FIB-4 is calculated by: [age (years) x AST (U/L)] / [platelet count (10^9^/L) x (ALT (U/L))^½^] [11]. A cut-off of < 1.45 is able to exclude advanced fibrosis with a negative predictive value of 90% and a sensitivity of 70%, while a cut-off of > 3.25 has a positive predictive value of 65% with a specificity of 97% in predicting fibrosis (AUROC =0.77).

### NAFLD fibrosis score

The NAFLD fibrosis score (NFS) consists of the variables age, BMI, diabetes, AST/ALT ratio (De Ritis ratio), platelet count and serum albumin. It is calculated as follows: NFS = − 1.675 + 0.037 x age (years) + 0.094 x BMI (kg/m^2^) + 1.13 * IGF/diabetes (yes = 1, no = 0) + 0.99 x AST/ALT ratio – 0.013 x platelet (× 10^9^/l) – 0.66 x albumin (g/dl). Results can be positive, negative or intermediate and predict the absence or presence of significant fibrosis with two cut-off points (− 1.46 and 0.68) [14]. A low cut-off score of − 1.46 is able to exclude advanced fibrosis with a negative predictive value of 88%, while a high cut-off score of 0.68 diagnoses advanced fibrosis with a positive predictive value of 82% (AUROC = 0.82).

### Statistical analysis

Graph Pad Prism® v7 was used for statistical analysis. Values are reported as mean and standard deviation (SD) or median and interquartile range (IQR) unless otherwise indicated. ANOVA and the two-sided (students) t-test were used for calculation of statistical significance. A two-sided *p* < 0.05 was considered statistically significant. Receiver operating characteristic (ROC) analysis was performed to analyze sensitivity and specificity.

## Results

### Patients’ characteristics

One hundred forty-one patients were included in this study between 2013 and 2018. See Table 1 for detailed clinical and demographic data.

**Table 1:** Patients’ characteristics

**Demographic data**		
Male	38	27%
Female	103	73%
Age (years)	44	± 9
BMI (kg/m^2^)	53	± 7
**Comorbidities**		
Type 2 diabetes mellitus	50	35%
Arterial hypertension	86	61%
Obstructive sleep apnea syndrome	58	41%
**NAFLD Activity Score (NAS)**		
Not NASH	49	35%
Borderline	65	46%
Definite NASH	27	19%
**Steatosis-activity-fibrosis (SAF) Score**		
No NAFLD	24	17%
NAFLD	59	42%
NASH	58	41%

Of the 141 patients, more than 70% were female (*n* = 103). Around half of the patients underwent gastric bypass procedure (*n* = 72, 51%), the other half received sleeve gastrectomy (*n* = 67, 48%). Two patients received a mini-bypass (1%). The average age of all patients was 43 ± 9 years. The mean body mass index was 53 ± 7 kg/m^2^. More than half of the patients had arterial hypertension (*n* = 86, 61%). Obstructive sleep apnea syndrome (OSAS) and type 2 diabetes mellitus were present in 41 and 35% of patients, respectively.

### Liver histology

**Fig. 1:**
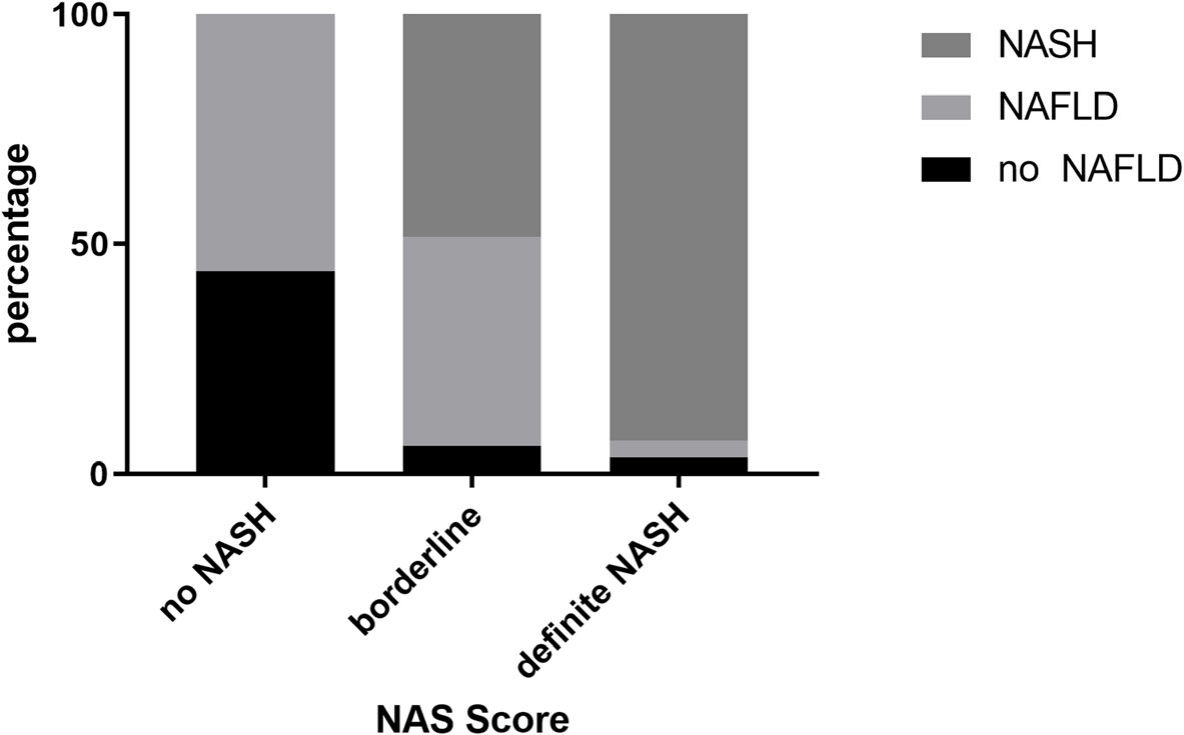
Comparison of NAS and SAF score 56% of the patients with no NASH in NAS were diagnosed as NAFLD in SAF score. 48% of the patients classified as borderline in NAS were identified as NASH in SAF score. Abbreviations: SAF: steatosis, activity, fibrosis score, NAS: NAFLD activity score

According to NAFLD activity score (NAS), ‘not NASH’ was observed in 35% of patients (*n* = 49), 46% of patients were classified as ‘borderline’ (*n* = 65) and 19% (*n* = 27) were classified as ‘definite NASH’. Median NAS score was 3 (IQR 3). According to SAF score, 24 (17%) showed ‘no signs of NALFD’, 59 patients (42%) were classified as ‘NAFLD’ and 58 (41%) as ‘NASH’ (Table 1). Patients with ‘no NASH’ in NAS were classified as ‘no NAFLD’ (44%) or ‘NAFLD’ (56%) in SAF. Patients with ‘no NAFLD’ according to SAF were classified as ‘not NASH’ (88%) and ‘borderline NASH’ (12%) in NAS score. Out of the patients with ‘borderline NASH’ in NAS, 48% were classified as ‘definite NASH’ by SAF score (Fig. 1).

Thirty-nine patients (28%) had a no fibrosis (F0), 61 patients (43%) had a stage 1 fibrosis (F1), 29 patients (21%) a stage 2 fibrosis (F2) and 12 patients (8%) a stage 3 fibrosis (F3). None of our patients showed a manifest cirrhosis (F4).

A mean of 25 portal tracts per sample was analysed (SD 8).

### NASH clinical scoring system

**Fig. 2:**
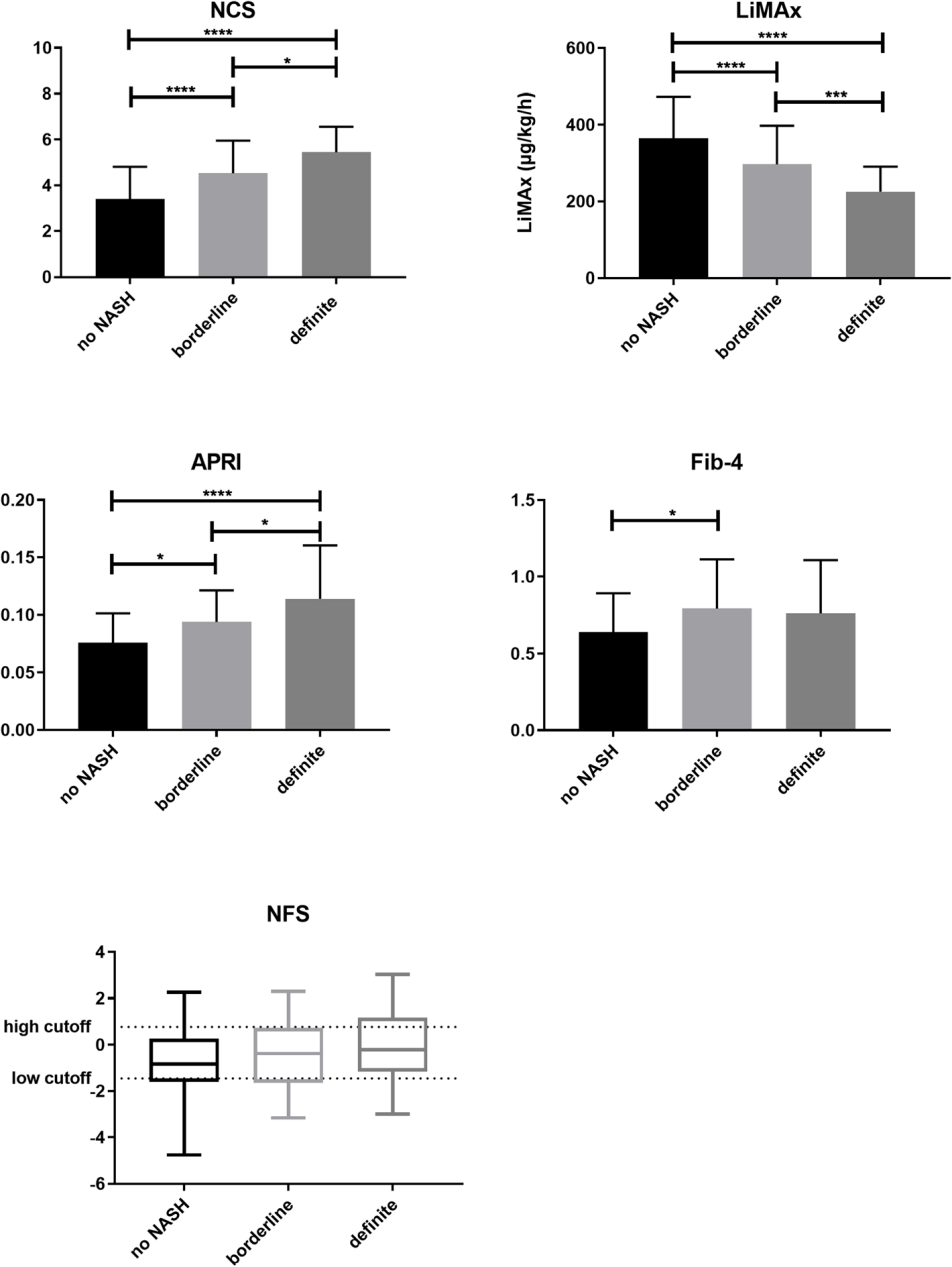
NAS score in comparison to clinical scores (*p*-values **** < 0.0001; *** < 0.001; * < 0.05); Abbreviations: NAS: NAFLD activity score, NCS: NASH Clinical Scoring System, LiMAx: LiMAx liver function capacity test, APRI: aspartate aminotransferase to platelet ratio index, NFS: NAFLD fibrosis score

Median sum score for ‘not NASH’ was 3 (IQR 3), for borderline 5 (IQR 1) and for manifest NASH 6 (IQR 1) (Fig. 2).

In distinguishing ‘NASH‘from ‘no NAFLD‘, a sensitivity of 62% and a specificity of 72% for SAF score was calculated (AUROC 0.77, cut-off value 5).

Patients with F0 to F2 fibrosis scored a median of 4, whereas patients with stage 3 fibrosis scored a median of 6 (Fig. 4).

In distinguishing no fibrosis from stage 3 fibrosis, a score of 6 revealed a sensitivity of 75% and a specificity of 91% (AUROC 0.85).

### Liver function capacity

LiMAx values were significantly higher in patients without NASH than in patients with ‘definite NASH‘(365 vs. 225 μg/kg/h; *p* < 0.0001) (Fig. 2). For NAS, sensitivity and specificity of LiMAx test to identify ‘definite NASH’ from ‘not NASH’ was 80 and 83% respectively (AUROC 0.88, cut-off value 263 μg/kg/h). For SAF, sensitivity and specificity were 79 and 82% (AUROC 0.87, with a cut-off value of 296 μg/kg/h). Liver function capacity showed no statistically significant differences between the various fibrosis stages (Fig. 4).

### APRI

**Fig. 3:**
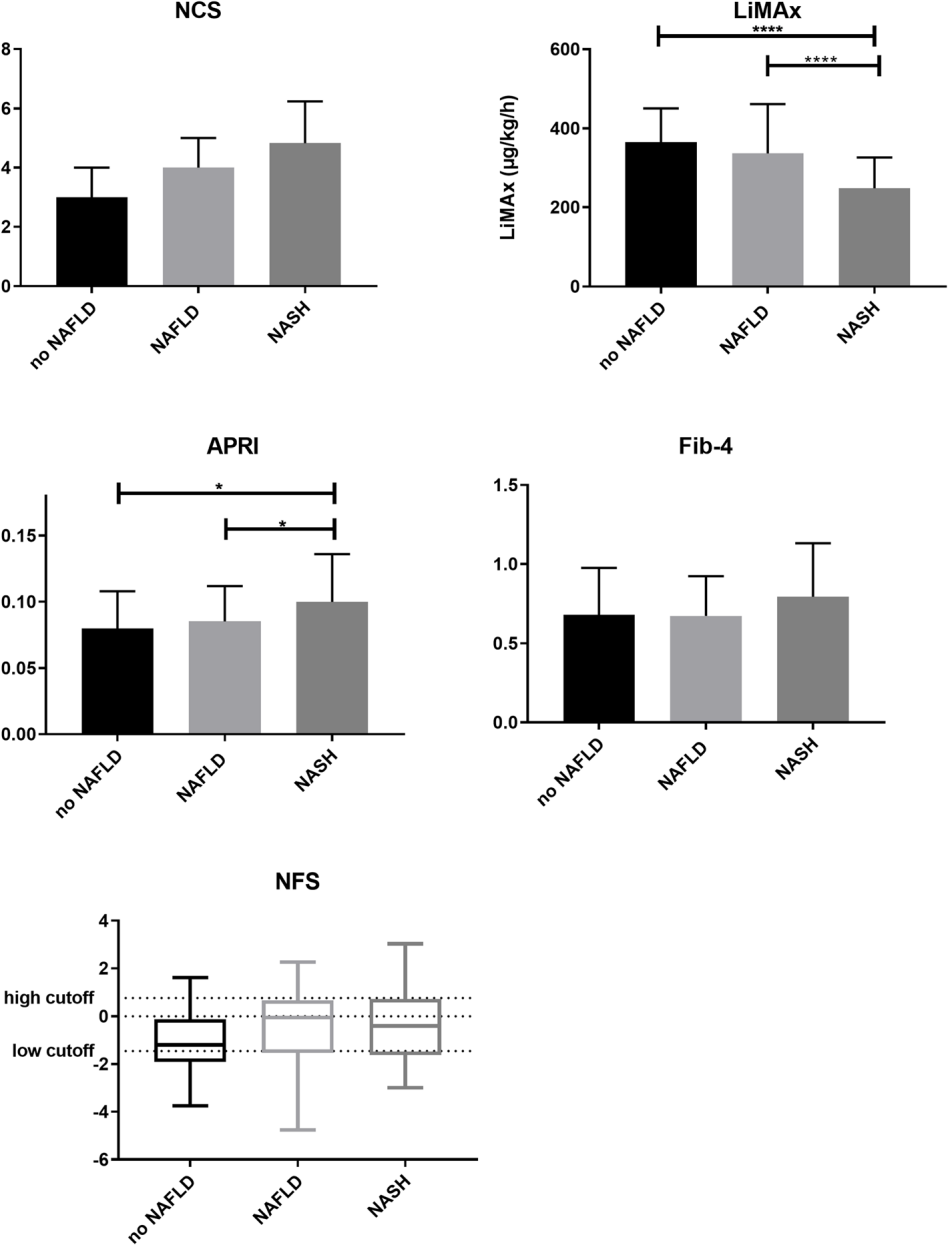
SAF score in comparison to clinical scores (*p*-values **** < 0.0001; *** < 0.001; * < 0.05); Abbreviations: SAF: Steatosis, Activity, Fibrosis Score, NCS: NASH Clinical Scoring System, LiMAx: LiMAx liver function capacity test, APRI: aspartate aminotransferase to platelet ratio index, NFS: NAFLD fibrosis score

**Fig. 4:**
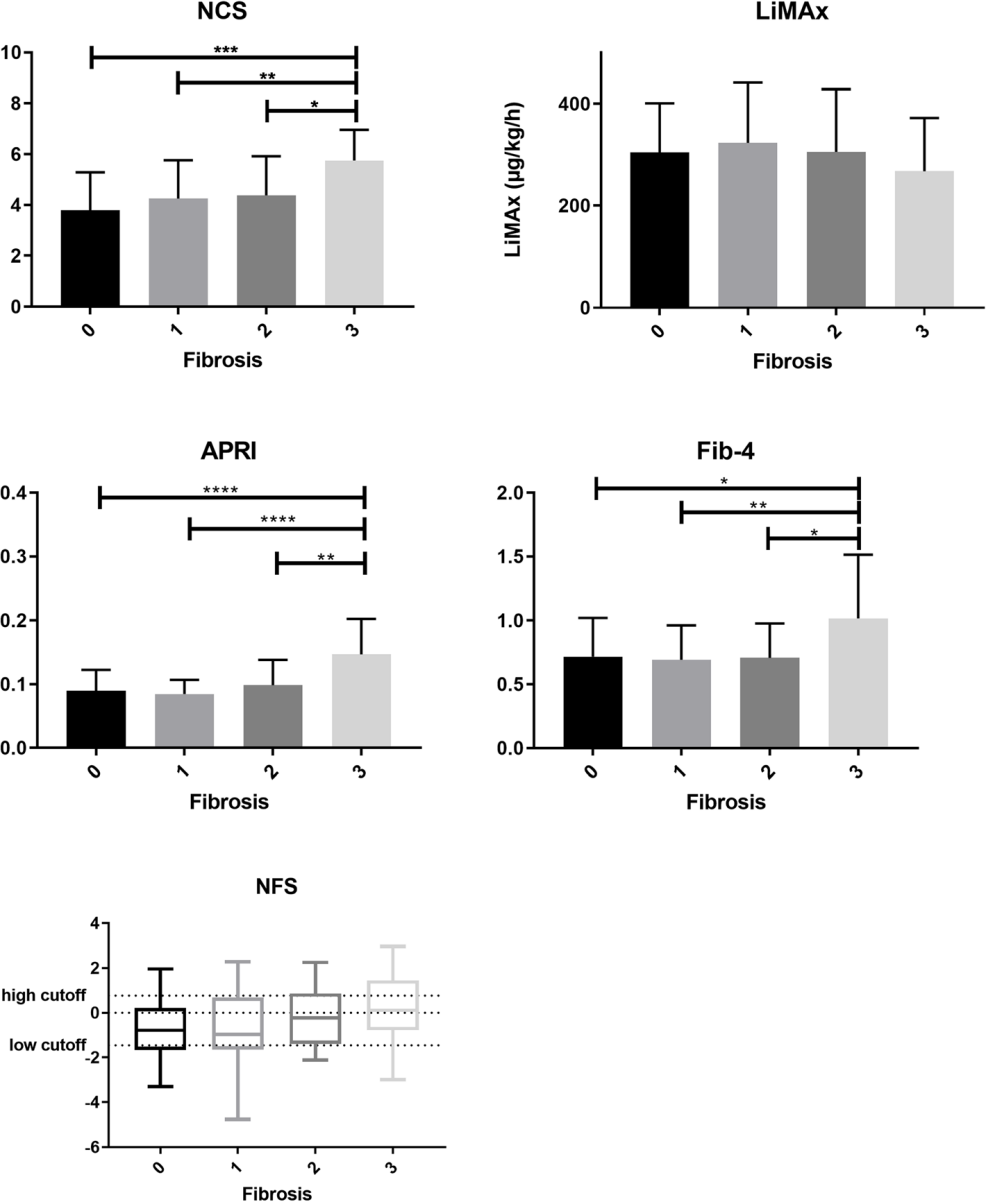
Fibrosis stages in comparison to clinical scores (*p*-values **** < 0.0001; *** < 0.001; ** < 0.01; * < 0.05), Abbreviations: NCS: NASH Clinical Scoring System, LiMAx: LiMAx liver function capacity test, APRI: aspartate aminotransferase to platelet ratio index, NFS: NAFLD fibrosis score

Mean APRI was 0.08 in patients with ‘not NASH’ and 0.12 in patients with ‘definite NASH‘(*p* < 0.0001) (Fig. 2). For a cut off-value of > 0.08, a sensitivity of 74% and specificity of 67% were calculated in distinguishing ‘not NASH’ from ‘definite NASH‘(AUROC 0.76). Using the SAF score, APRI also showed significant differences between ‘no NAFLD‘and ‘NASH‘(0.08 versus 0.1, *p* = 0.02) (Fig. 3). APRI reached herein a sensitivity of 63% and a specificity of 65% (AUROC 0.67) and was furthermore able to show significant differences between the individual stages of fibrosis (Fig. 4). Sensitivity and specificity to differentiate no fibrosis from stage 3 fibrosis was 83 and 76% for a cut-off value of 0.1 (AUROC 0.81).

### Fib-4

Fib-4 did not reach statistical significance discriminating between ‘not NASH’ and ‘NASH‘(Figs. 2 and 3). Statistical significance was only found concerning fibrosis (Fig. 4) with a sensitivity of 49% and a specificity of 65% (cut-off 0.78, AUROC 0.67).

### NAFLD fibrosis score

There was no difference between patients with ‘not NASH’ and ‘definite NASH’ neither for NAS, nor for SAF score (AUROC 0.63 and 0.62) (Fig. 2 and Fig. 3). NAFLD fibrosis score did not reveal any significant differences between fibrosis stages (Fig. 4).

For a summary of all scores used, see Table 2.

**Table 2:** Score overview

	NAFLD Activity Score (NAS) (no NASH – definite NASH)					Steatosis, Activity, Fibrosis Score (SAF) (no NAFLD – NASH)					Fibrosis (no fibrosis – Stage 3 fibrosis)				
	Cut-off	AUROC	SENS %	SPEC %	***p***-value	Cut-off	AUROC	SENS %	SPEC %	***p***-value	Cut-off	AUROC	SENS %	SPEC %	***p***-value
**NCS**	4	0.86	78	75	< 0.0001	5	0.77	62	72	< 0,0001	6	0.85	75	91	0.0002
**LiMAx**	263	0.88	80	83	< 0.0001	296	0.87	79	82	< 0.0001	292	0.53	55	58	0.72
**APRI**	0.08	0.76	74	67	< 0.0001	0.08	0.67	63	65	0.02	0.1	0.81	83	76	< 0.0001
**Fib-4**	0.67	0.58	53	54	0.25	0.71	0.57	51	54	0.36	0.78	0.67	49	65	0.02
**NFS**	−0.47	0.63	63	61	0.07	−1.02	0.62	59	58	0.09	−0.44	0.68	55	62	0.11

*Abbreviations*: *NAS* NAFLD Activity Score, *SAF* Steatosis, Activity, Fibrosis Score, *NASH* NASH: non-alcoholic steatohepatitis, *NAFLD* non-alcoholic fatty liver disease, *AUROC* Area under the Receiver Operating Characteristic, *SENS* Sensitivity, *SPEC* Specificity, *NCS* NASH Clinical Scoring System, *LiMAx* LiMAx liver function capacity test, *APRI* aspartate aminotransferase to platelet ratio index, *NFS* NAFLD fibrosis score

## Discussion

NAFLD and its progressive form NASH are emerging conditions especially among obese patients. Gold standard for diagnosis is liver biopsy with subsequent histopathological evaluation. The most common histological scoring systems for NAFLD are the NAFLD activity score (NAS) and the steatosis-activity-fibrosis (SAF) score [21, 24]. In this study, NAS and SAF showed a high concordance in diagnosing definite NASH, whereas nearly half of the patients classified as ‘borderline‘by NAS had a ‘definite NASH‘according to SAF score. This is in line with the findings of Rastogi et al., whose rate of cases underdiagnosed by NAS was even higher (88%). As Brunt et al. pointed out, the NAS was originally established for monitoring therapeutic effects, whereas the SAF score was initially designed to differentiate between NAFLD and NASH [10]. Thus, there might be a risk in missing patients with manifest NASH if diagnosis solely relies on NAS. Both scores should be applied where possible.

Concerning long term-outcome of NAFLD and NASH, the development of liver fibrosis appears to be the crucial parameter [25]. Liver fibrosis is included in SAF score by Bedossa et al., but analysed separately in NAS [21, 24]. When applying NAS for diagnosis of NAFLD and NASH, additional evaluation of liver fibrosis needs to be mandatory.

Liver biopsy certainly allows for definite grading and staging of NAFLD. The risks associated however do not justify routine clinical usage. This being additionally amplified by the high margin of sampling errors in transcutaneous biopsies [26, 27]. Different low-risk, non-invasive tests have therefore been proposed to avoid liver biopsy, but up to now no single test has been established in clinical practice. In this study, we were able to obtain liver biopsies of obese subjects with NAFLD intraoperatively, resulting in larger specimens with a lower sampling error [28]. Out of the clinical scoring tests applied in this study, APRI proved most useful, as it was the only test able to securely differentiate between no NASH and NASH as well as identify no fibrosis from severe fibrosis. This is especially remarkable, as it was initially designed to diagnose advanced fibrosis in patients with chronic virologic liver injury and not for diagnosing NAFLD or NASH [16, 18]. The usage of APRI has since widened and there have been successful validations of its use in non-alcoholic chronic liver diseases [16] as well as descriptions of its usage in monitoring hepatitis B under therapy [29] or diagnosis of fibrosis in post-hepatitis C patients [30]. AASLD practice guidelines also recommend its usage in diagnosis of NAFLD and NASH in obese patients [17].

Of the other non-invasive scores evaluated in this study, NASH clinical scoring system (NCS) also correlated well with the presence or absence of NASH according to NAS but did not show statistical significance in SAF score. NCS requires diagnosis of sleep apnea and it has to be taken in mind, that there are different ways to diagnose sleep apnea. Therefore, under- or overestimation of sleep apnea with consequently altered NCS scores might affect its results. Our group has already been able to describe the liver function capacity test (LiMAx) used in this study to aptly evaluate liver function in obese subjects [19, 31]. It now also performed well in differentiating no NASH from NASH, but failed to correctly predict liver fibrosis. Compared with APRI and the NASH clinical scoring system, it is not as regularly available and more cost-intensive. The LiMAx test has to be performed on a fasting patient and smoking might alter the results. Therefore, there is a risk of misclassification when performed under non-fasting conditions or on a patient with a history of smoking.

Fib-4, that was designed for detection of fibrosis in patients with hepatitis C and HIV [11], was insufficient in differentiating no NASH from NASH, but reached statistical significance in diagnosing liver fibrosis. Nevertheless, AUROC for discriminating liver fibrosis stages was lower than in APRI and routine calculation is more complex.

Interestingly, NAFLD fibrosis score was unable to diagnose NASH by any of the two histological scores applied and was furthermore unable to diagnose liver fibrosis. We therefore do not advocate its routine usage in contrast to AASLD practice guidelines. In this study, APRI proved vastly superior in diagnosing NASH and fibrosis in obese patients and performed better than Fib-4 and NAFLD fibrosis score. These findings concur with other studies, in which APRI also reached a higher accuracy than Fib-4 and NAFLD fibrosis score [32, 33]. APRI furthermore has the obvious advantage to be easily assessable and moderate in costs. It therefore could also be successfully applied in a low-income population [33].

For all non-invasive scores requiring laboratory values it has to be taken into account, that laboratory values may be divergent from day to day and therefore create different results.

In our bariatric center, all patients with definite NASH in liver histology are referred to a gastroenterologist / hepatologist. Patients with borderline NASH undergo control of laboratory values and re-calculation of clinical scores. If there is no improvement, a control biopsy is considered. Patients without NASH receive routine laboratory monitoring after bariatric surgery.

A limitation of this study is the relatively small number of 141 obese patients included. There is certainly a selection bias, as all of the participants underwent bariatric surgery. Cut-off values in this study therefore refer to morbidly obese subjects and might not be applicable to the general population. Furthermore, data was collected prospectively but analysis was carried out in a retrospective manner. Nevertheless, prevention and handling of NAFLD are gaining importance in an increasingly overweight global populace. Therefore, a simple score such as the APRI described in this study might facilitate selecting patients at risk for NAFLD or in need of treatment.

## Conclusion

NASH clinical scoring system and LiMAx liver function capacity test were useful in discriminating NASH from no NASH but failed in correctly predicting liver fibrosis. Fib-4 was futile in diagnosing NASH but had its usage in diagnosing liver fibrosis, whereas the NAFLD fibrosis score proved neither sufficient for diagnosis of NASH, nor liver fibrosis. APRI showed a high accuracy in detecting NASH and advanced liver fibrosis in obese patients. The easy-to-assess, easy-to-access APRI could therefore be used for deciding which patients to select for a liver biopsy during bariatric surgery.

Comparing the histologic scores applied in this study, the NAS score might underdiagnose certain patients with NASH by classifying them as borderline.

## Data Availability

The datasets used and/or analysed during the current study are available from the corresponding author on reasonable request.

## Abbreviations

ALT: Alanine aminotransferase
APRI: Aspartate aminotransferase to platelet ratio index
AST: Aspartate aminotransferase
AUROC: Area under the receiver operating characteristic
BMI: Body mass index
Fib-4: Fib-4 Index
IQR: Interquartile range
LiMAx: LiMAx liver function capacity test
NAFLD: Non-alcoholic fatty liver disease
NAS: NAFLD activity score
NASH: Non-alcoholic steatohepatitis
NCS: NASH Clinical Scoring System
NFS: NAFLD fibrosis score
ROC: Receiver operating characteristic
SAF: Steatosis, activity and fibrosis score
SENS: Sensitivity
SPEC: Specificity
